# The Effects of Pre-Storage Leukoreduction on the Conservation of Bovine Whole Blood in Plastic Bags

**DOI:** 10.3390/biology9120444

**Published:** 2020-12-04

**Authors:** Brena Peleja Vinholte, Rejane dos Santos Sousa, Francisco Flávio Vieira Assis, Osvaldo Gato Nunes Neto, Juliana Machado Portela, Gilson Andrey Siqueira Pinto, Enrico Lippi Ortolani, Fernando José Benesi, Raimundo Alves Barrêto Júnior, Antonio Humberto Hamad Minervino

**Affiliations:** 1 Laboratory of Animal Health, LARSANA, Federal University of Western Pará, UFOPA, Rua Vera Paz, s/n Bairro Salé, Santarém 68040-255, PA, Brazil; brenavinholte@hotmail.com (B.P.V.); flavioassis131995@hotmail.com (F.F.V.A.); vet.osvaldo@gmail.com (O.G.N.N.); enf_julianamachado@hotmail.com (J.M.P.); gilsonandrey@gmail.com (G.A.S.P.); 2 Department of Clinical Science, College of Veterinary Medicine and Animal Science, University of Sao Paulo (FMVZ/USP, Av. Prof. Dr. Orlando Marques de Paiva, 87, Cidade Universitária, Butantã, São Paulo 05508-270, SP, Brazil; rejane.santossousa@gmail.com (R.d.S.S.); ortolani@usp.br (E.L.O.); febencli@usp.br (F.J.B.); 3 Department of Animal Science, Federal Rural University of the Semiarid Region, UFERSA, Av. Francisco Mota, 572-Bairro Costa e Silva, Mossoró 59625-900, RN, Brazil; barreto@ufersa.edu.br

**Keywords:** cattle, blood storage, transfusion reactions, white blood cells

## Abstract

**Simple Summary:**

Blood transfusion is a life-saving veterinary therapeutic procedure. While fractionated blood components are used in humans, whole blood is most commonly used in animals, especially for farm animals. Whole blood contains white blood cells that can cause a transfusion reaction in animals. Here, we proposed that using a blood bag with leukocyte filtration is sufficient for blood conservation under field conditions and thus can be an option for transfusion medicine in the case of farm animals. The filtered bag was efficient in removing white cells from cattle whole blood and could be used under field conditions. Blood stored after white blood cells were removed showed less acidic load. Further experimental studies are required to prove that blood without white cells results in a decrease in transfusion reactions in cattle.

**Abstract:**

Leukoreduction (LR) is a technique that consists of reducing the number of leukocytes in whole blood or blood components that can contribute to decreasing storage lesions and the occurrence of post-transfusion complications. We propose that using a blood bag with pre-storage leukocyte filtration is sufficient for blood conservation under field conditions. Ten healthy Nelore cows were used. Whole blood was sampled from each animal and stored at 2 to 6 °C in CPD/SAG-M (citrate phosphate dextrose bag with a saline, adenine, glucose, mannitol satellite bag) triple bags (Control) and in CPD/SAG-M quadruple bags with a leukocyte filter (Filter). At baseline and after 7, 14, 21, 28, 35, and 42 days (D0, D7, D14, D21, D28, D35, and D42, respectively), complete hematological, blood gas, and biochemical evaluations were determined. The filtered bag removed 99.3% of white blood cells from cattle blood, and the entire filtration process was performed in the field. There was a reduction in the number of red blood cells (RBCs) in both groups from D14 onward, with a decrease of 19.7% and 17.1% at D42 for the Control and Filter bags, respectively. The hemoglobin (Hb) concentration had variation in both groups. Potassium, pO_2_, pCO_2_, and sO_2_ increased, and sodium, bicarbonate, and pH decreased during storage. The filtered bag was efficient in removing white cells from cattle whole blood and could be used under field conditions. Blood stored after LR showed differences (*p* < 0.05) in blood gas analysis towards a better quality of stored blood (e.g., higher pH, lower pCO_2_, higher sO_2_). Further experimental studies are required to prove that blood without white cells results in a decrease in transfusion reactions in cattle.

## 1. Introduction

The development of different preservative solutions is essential for the long-term storage of blood, especially to facilitate the use of blood transfusion in human and animal medicine. However, stored whole blood components undergo changes known as storage lesions [1,2]. The leukocyte degradation in the storage blood results in the release of cytokines, histamine, serotonin, elastase, and acid phosphatase that contribute to hemolysis and post-transfusion complications [3,4,5].

Leukoreduction (LR) consists of reducing the number of leukocytes in whole blood or blood components such as red blood cell (RBC) concentrate, platelet concentrate, or plasma. LR has been useful in decreasing febrile non-hemolytic transfusion reaction (FNHTR), and decreasing the mortality of patients undergoing cardiac surgery [6,7,8,9,10]. In veterinary medicine, the majority of studies have been performed in dogs, in which LR resulted in a 98% leukocyte and 95.1% platelet count reduction [11]. LR in dogs’ blood was effective in preventing the release of vascular endothelial growth factor [12], preventing the increase in interleukin-8 concentration [13], and attenuating the generation of phosphatidylsering-expressing microparticles [14] during storage. LR eliminated the post-transfusion inflammatory response in dogs receiving stored packed red blood cells [15].

Although blood fractionation techniques are used to obtain individual blood components, the conservation and use of whole blood is still the most used method [16,17,18] in large animal clinics where it is more difficult to use fractionated blood. At the field level, it is necessary to invest in more infrastructure and equipment to perform fractionation. Due to the difficulty of performing fractionation in the field, the use of a leukocyte filtering bag would advance blood conservation and transfusion therapy for large animals, as it would allow for the removal of leukocytes immediately after blood collection, without the need for a field-level centrifuge. We propose that using a human blood bag with pre-storage leukocyte filtration will improve blood quality for conservation purposes under field conditions, being a novel option for veterinary transfusion medicine.

## 2. Materials and Methods

This study was approved by the Ethics Commission of Animal Use of the Federal University of Western Pará, Santarém, PA, Brazil, protocol number 01002-2016. Ten adult female Nellore cattle were used, weighing an average of 406.5 ± 42.69 kg each. Animals were healthy under physical examination, had normal hematological parameters, and had negative smears to blood parasites (*Babesia* sp., *Anaplasma* sp., and *Trypanossoma* sp.). For blood sampling, cattle were placed in a cattle crush. Complete fur removal and antisepsis were performed in the neck region over the jugular vein using povidone iodine and then 70% alcohol. The reason for the sample size of ten animals was because the same animal could provide blood for the two bags, which was collected at once, thus reducing the variability between groups.

From each animal, 900 g of blood was sampled, 450 g of which was stored in a CPD/SAG-M triple bag (CompoSampler; Fresenius Kabi, São Paulo, Brazil), and 450 g was stored in a CPD/SAG-M quadruple bag with an in-line leukocyte filter (CompoFlow^®^ Select; Fresenius Kabi, São Paulo, Brazil), comprising the Control and Filter groups, respectively. Both bags contained a preservative solution composed of citrate, phosphate, and dextrose in the primary bag, and a solution of mannitol and sodium chloride in the satellite bag. Only one venipuncture was performed for blood collection, the blood being stored in one type of bag until full, which was then exchanged for the other type, using the same venous access. The order that defined the type of bag in the sampling sequence (first or second) was alternated for each animal.

For the Filter group, following the manufacturer’s instructions, pre-storage filtration for leukocyte removal was performed one hour after sampling and prior to mixing in the additive from the satellite bag. For the filtration, we used the provided blood bags moving the whole blood using gravidity from one bag to another through an in-line filter. The filtration time varied from 15 to 30 min, after which the additive solution (from the satellite bag) was mixed into the filtrate, homogenized, and stored in the refrigerator. The Control group followed previously described procedures [19]. The bags were stored in a refrigerator with a controlled temperature of 2 to 6 °C. The refrigerator was placed in the lab and was restricted for this experiment. The blood bags were homogenized every two day. Around 45 mL of blood was lost in the white blood filter, with a red blood recovery of 90% using this in-line filtration system.

Laboratory evaluations of the filtered and whole blood stored in the bags were performed at seven different times as follows: immediately after collection (D0), 7 days after collection (D7), 14 days after collection (D14), 21 days after collection (D21), 28 days after collection (D28), 35 days after collection (D35), and 42 days after collection (D42).

Prior to the evaluation of the blood stored in the bags, the blood was homogenized for 10 to 20 min, followed by the withdrawal of 15 mL of blood using sterile syringes to measure the hematological, blood gas, and biochemical variables. In addition, a microbiological examination was performed at only D0 and D42.

The red and white blood cell counts were determined manually in a Neubauer chamber with a macrodilution technique [20]. The platelet count was not performed. The packed cell volume (PCV) was obtained using a microhematocrit centrifuge, and total hemoglobin (Hb) through the cyanmethemoglobin method [21]. The mean corpuscular cell volume (MCV) was calculated using the equation described by Jain [22]. Measurements of pH, partial O_2_ pressure (pO_2_), partial CO_2_ pressure (pCO_2_), saturation of O_2_ (sO_2_), and bicarbonate (HCO_3_) values were performed using a portable blood gas analysis device (i-STAT^®^ System, Abbott Laboratories, USA) using commercial cartridges (CG8+, Abbott Laboratories, Chicago, IL, USA).

Whole blood samples were centrifuged under refrigeration (4 °C) for 10 min at 1000× *g* to obtain plasma for the assessment of lactate, glucose, cholesterol, sodium, and potassium concentration using an automatic biochemical analyzer (Rx Daytona, Randox, Antrim, UK) with commercial kits (Randox), with the exception of sodium and potassium which were determined using a flame photometer (CELM, São Paulo, Brazil).

The Hemobac Triphasic System (ProBac do Brasil, São Paulo, Brazil) was used for microbiological analysis, with the culture media (chocolate agar, Sabouraud’s agar, and MacConkey’s agar) being kept in an incubator for seven days at a temperature of 35 °C.

Data were assessed for Gauss distribution using the Kolmogorov–Smirnov test. Data showing normal distribution were subjected to analysis of variance (ANOVA) using the PROC MIXED procedure of SAS (Statistical Analysis System, SAS Institute Inc., Cary, NC, USA) for repeated measures over time, considering the effects of treatment (Control and Filter bags), time (different time points), and the interaction of time and treatment. The Bonferroni test was used to determine the differences between D0 and each of the other time points. A *P*-level of 0.05 was considered statistically significant.

## 3. Results

There was no microbiological contamination at D0 or at the end of the study according to the results from the Hemobac culture test. The blood bag with the leukocyte filter proved to be efficient, removing 99.3% of the white cells from cattle blood, with the entire filtration process being performed in the field. The mean number of leukocytes before filtration was 12.1 ± 1.0 × 10^3^, which was reduced to 0.068 ± 0.01 × 10^3^ after filtration using the in-line system in the storage bag. The filter was efficient at removing white cells by 99.3%. Although we did not count platelets, stored blood after LR had probably a marked decrease in platelet count.

There was no difference between the Control and Filter groups for the variables of RBC, Hb, and PCV (Table 1). There was a decrease in the total number of RBCs in both groups, from D14 to D42. There was no change in PCV over time. Hb increased in both bags at D14 and onwards. There was no difference between the Control and Filter groups for the MCV; however, there was an increase in MCV for the Control group after D35.

Higher pH and concentrations of pCO_2_, pO_2_, and sO_2_ were observed in the blood stored in the filtered bags as compared to the control bags (Table 2). When comparing D0 with the other time points, there was a reduction in blood pH in the Control group after D14, whereas in the Filter group there was a reduction only at D42. Only the Control group stock showed variations in pCO_2_ in relation to time, with an increase starting at D21.

In relation to pO_2_, there was an increase after D28 and D14, in the Control and Filter groups, respectively. The values of sO_2_ increased for both groups from D14 onward. For bicarbonate, the Control group decreased after D7, and the Filter group after D14.

There was no difference between the groups for the concentrations of glucose, cholesterol, lactate, potassium, or sodium (Table 3). However, when comparing D0 with the other time points, glucose decreased in both groups after D28, and potassium increased and sodium decreased in concentration after D7. Lactate increased in concentration after D14 in both groups, whereas no difference was observed over time for cholesterol.

## 4. Discussion

The filtered bag proved that it can be used under farm conditions, as this experiment was performed in a commercial farm and none of the samples had microbiological contamination. Greenwalt et al. [23] and Heaton et al. [24] state that the presence of leukocytes in the bags of RBCs contributes significantly to an increase in hemolysis during storage, mainly due to the release of various chemicals and enzymes, but especially leukocyte proteases. However, the absence of a difference in the number of RBCs and in the concentration of Hb between the bags suggests that the filtered bag did not confer additional benefits in the preservation of erythrocytes in bovine species when compared to the triple bag without leukoreduction.

Nunes Neto et al. [19] working with buffalo whole blood described that PCV showed no differences during storage, but showed differences between types of bags due to the differences in preservative solutions. In this study, as the bags used had the same conservative solution and volume, the absence of differences between the bags is justified. Despite the reduction in the number of RBCs in the stored whole blood, the PCV remained stable due to the increase in the MCV as observed in other studies [25,26]. Regarding total Hb, Tavares et al. [18] and Barros [27] found similar results, with significant differences between volumes, and an increase between time points.

In the filtered bags, the number of leukocytes before and after filtration was compared at D0, where the statistical difference is evident, showing that the filter was efficient at removing white cells by 99.3%. Moreover, we did not perform a platelet count; for human blood, using the same blood bag with the in-line filter removes ~97% of platelets, therefore we can assume that the filtered blood had a substantial reduction in platelet count [28]. Further studies with cattle blood should confirm this assumption. Studies show that the leukoreduction of blood components has been a viable and effective resource in reducing the occurrence of transfusion incidents. However, the filters used in this procedure must be able to remove at least 90% of the leukocytes present in the RBC concentrate so that the risks associated with transfusions are reduced [29].

In this study, there was a reduction in the number of leukocytes in the control bags during storage, which is compatible with previous studies in different species [17,18,19]. However, even after 42 days of storage, the blood had a significative amount of leukocytes, which can contribute to a higher occurrence of post-transfusion febrile reaction [6,30]. Other studies indicate that the presence of leukocytes can have indirect deleterious effects on stored erythrocytes, as these cells contribute to the consumption of glucose from the conservative solution and also release bioreactive substances. During storage, these cells rupture, promoting the release of immunomodulators. Therefore, their presence in stored blood products can accelerate hemolysis and increase extracellular potassium [15,31]. Thus, the blood from the filtered bags had those advantages when used for transfusion in cattle.

The pH reduction was more accentuated in the control bags, occurring due to the production of acid metabolites, such as lactate, by the stored red cells [17,32,33]. Although the pH reduction is related to the degradation of 2,3-diphosphoglycerate, in ruminants, this metabolite has no effect on the affinity of hemoglobin for O_2_, since the cattle Hb has a larger preferential binding with chorine ions [34]. This difference in pH between the bag types can be attributed to the absence of leukocytes, as the Control group showed a greater pH reduction and the Filter group showed less variation. Additionally, the presence of leukocytes can increase the consumption of glucose and consequently the production of lactate, influencing the pH drop in the control bags. Although there was no difference between the bags for glucose and lactate, it is evident that the control bags presented a greater variation of these variables throughout the study. However, another factor that may have contributed to blood acidification is pCO_2_, which was higher in the control bags.

The increase in pCO_2_ in stored blood is mainly due to the neutralization of lactic acid produced by cellular metabolism, resulting in the production of CO_2_. The increase in pCO_2_ is another factor that causes a decrease in Hb affinity through two mechanisms—first, by decreasing blood pH and, second, by promoting a direct combination of CO_2_ with Hb-forming carbamino compounds [35].

PO_2_ was higher in the blood stored in the filtered bags, but increased in both bags over time. The increase in pO_2_ during storage has been observed in the stored blood of canines, bovines, sheep, and donkeys [17,27,33,36]. Like pO_2_, sO_2_ was higher in the filtered bags. The affinity of O_2_ to Hb can be represented by a Hb dissociation curve in which the higher the partial O_2_ pressure values, the higher the O_2_ saturation values [25].

Moroz [37] showed that the leukoreduced RBC concentrate in dogs had higher saturation and O_2_ pressure both in the blood stored in CPDA-1 and CPD/SAG-M bags when compared with non-leukoreduced concentrate in the same types of bags. This is similar to the results found in our study with cattle whole blood.

The reduction in blood values of HCO_3_ occurs due to its consumption of lactate in the control of acidity [38]. Ribeiro Filho et al. [32] associates the gradual reduction of HCO_3_ levels with the increase in lactate, in order to neutralize this acid. The limited variation in cholesterol in the storage period can be explained by Roback et al. [39], where they suggest that over the conservation period, erythrocytes, when aging, make repairs to their membranes, preferably using phospholipids such as phosphoglycerol, with cholesterol remaining with its concentrations unchanged. Cholesterol contributes to the stabilization and fluidity of the erythrocyte plasma membrane. The data found corroborate the findings of Nunes Neto et al. [19], who also found no variation in the cholesterol of buffalo blood during 42 days of storage.

The increase in lactate levels during blood storage is consistent with and described in the blood conservation assessments of humans [39], donkeys [27], sheep [17], and buffalo [19]. During conservation, the glucose consumed to produce ATP generates a series of metabolites, including lactate. This process, called anaerobic glycolysis, is an alternative mechanism for energy production in tissues with insufficient amounts of O_2_ or in cells without mitochondria such as RBCs [39]. Kaneko et al. [40] describes that, during anaerobic glycolysis, two lactate molecules are produced for each metabolized glucose molecule. The consequences of the increase in lactate are related to the decrease in pH levels during the conservation period, and rapid blood infusions or transfusions to seriously injured patients should be avoided in order to avoid acidemia [41].

High values of potassium in the stored blood are dependent on hemolysis, and intra-erythrocyte potassium concentrations between species, with high concentrations found in the red cells of humans, horses, goats, and cattle, and low values for dogs and cats [25]. Like Hb, the increase in extracellular potassium concentration indicates one of the first events that are related to the reduction in the quality of red cells [42].

The bags with the leukocyte filter contributed to the better quality of the stored blood since they presented less acidity, which consequently influences greater efficiency in the release of O_2_ by Hb. However, further studies are needed to assess the accumulation of bioreactive substances such as cytokines, free radicals, and pro-inflammatory products, as well as their post-transfusion effects.

Another potential benefit of LR is related to the reduction of the transmission risk of infectious diseases through blood transfusion. Experimental studies in animal models have confirmed that LR provides a high, but not absolute, protection against prion disease transmission by blood transfusion [43]. Leukodepletion was beneficial in the removal of human pathogens such cytomegalovirus, *Orientia tsutsugamushi*, and *Trypanosoma cruzi*, and may reduce the Leishmania transmission hazard [44]. A recent study with dogs shows that LR reduced but did not eliminate *Rickettsia conorii* in stored whole blood [45]. Further studies are required to evaluate the effectiveness of LK in the transfusion-transmitted infectious diseases important to the cattle industry.

## 5. Conclusions

The whole blood of cattle stored in CPD/SAG-M triple bags and CPD/SAG-M quadruple bags with in-line leukocyte filtration changed during the storage period of 42 days under refrigeration; however, the blood remained viable for transfusion. The two types of bags evaluated can be indicated for conservation and transfusion of whole blood in the bovine species. In assessing the efficiency of the leukocyte filter, the Filter blood bag proved to be efficient, removing 99.3% of the white cells from bovine whole blood, and can be used under field conditions.

## Figures and Tables

**Table 1:** Mean values and standard deviations of the hematological variables of the cattle whole blood stored in CPD/SAG-M (Control (C)) and CPD/SAG-M with Filter (Filter (F)) bags for 42 days.

Variables	Bags	Times							*p*		
		D0	D7	D14	D21	D28	D35	D42	Treatment *	Time	Treat × Time
PCV (%)	C	23.5 ± 3.7	23.3 ± 3.9	22.0 ± 3.2	22.1 ± 3.2	21.5 ± 4.2	22.2 ± 4.3	22.1 ± 4.2	0.7484	0.0596	0.9872
	F	22.7 ± 4.4	22.6 ± 4.2	21.3 ± 3.3	21.1 ± 2.9	21.4 ± 3.7	21.0 ± 3.9	21.0 ± 3.8			
RBC (×10^6^)	C	7.6 ± 0.5 ^a^	7.2 ± 0.4 ^a^	7.0 ± 0.4 ^b^	7.0 ± 0.3 ^b^	6.3 ± 0.4 ^b^	6.1 ± 0.5 ^b^	6.0 ± 0.4 ^b^	0.9452	0.0001	0.0948
	F	7.6 ± 0.4 ^a^	7.4 ± 0.4 ^a^	6.6 ± 1.1 ^b^	6.6 ± 0.7 ^b^	6.4 ± 0.6 ^b^	6.3 ± 0.7 ^b^	6.2 ± 0.6 ^b^			
Total Hb (g/dL)	C	8.0 ± 1.4 ^a^	7.0 ± 1.1 ^ab^	6.4 ± 1.0 ^b^	7.6 ± 1.0 ^ab^	6.2 ± 0.9 ^b^	6.9 ± 0.9 ^ab^	7.1 ± 0.7 ^ab^	0.6702	0.0001	0.9999
	F	7.9 ± 1.4 ^a^	6.9 ± 1.1 ^ab^	6.2 ± 1.1 ^b^	7.4 ± 1.1 ^ab^	6.0 ± 1.0 ^b^	6.8 ± 0.8 ^ab^	6.9 ± 0.7 ^ab^			
MCV (fL)	C	31.1 ± 4.4 ^b^	32.1 ± 4.4 ^b^	31.6 ± 4.4 ^b^	32.6 ± 4.0 ^b^	33.9 ± 5.3 ^b^	36.1 ± 4.6 ^a^	36.1 ± 4.7 ^a^	0.4420	0.0005	0.7665
	F	29.7 ± 5.5	30.8 ± 6.3	31.9 ± 3.8	32.5 ± 6.3	33.6 ± 5.2	33.4 ± 3.8	33.4 ± 3.8			
WBC (×10^3^)	C	12.2 ± 1.0 ^a^	10.8 ± 1.5 ^b^	9.4 ± 1.1 ^b^	8.6 ± 1.1 ^b^	8.0 ± 1.1 ^b^	7.7 ± 0.8 ^b^	7.7 ± 0.8 ^b^	0.0001	0.0005	0.5643
	F	12.1 ± 1.1 ^a^	0.07 ± 0.07 ^b^	0.05 ± 0.02 ^b^	0.06 ± 0.01 ^b^	0.07 ± 0.01 ^b^	0.05 ± 0.01 ^b^	0.05 ± 0.01 ^b^			

* *p* < 0.05 indicates overall difference between the tested blood bags. Different lowercase letters on the line indicate a difference between times through Tukey’s paired comparison test. PCV: packed cell volume; RBC: red blood cell count; Hb: hemoglobin; WBC: white blood cell count. D: day. Treat × Time: treatment and time interaction.

**Table 2:** Mean values and standard deviations of the blood gas variables of the cattle whole blood stored in CPD/SAG-M (C) and CPD/SAG-M with Filter (F) bags for 42 days.

Variables	Bags	Times							*p*		
		D0	D7	D14	D21	D28	D35	D42	Treatment *	Time	Treat × Time
pH	C	6.96 ± 0.1 ^a^	6.83 ± 0.1 ^ab^	6.82 ± 0.1 ^b^	6.78 ± 0.1 ^b^	6.77 ± 0.1 ^b^	6.75 ± 0.1 ^b^	6.70 ± 0.1 ^b^	0.0002	0.0001	0.7268
	F	6.95 ± 0.1 ^a^	6.90 ± 0.1 ^ab^	6.87 ± 0.1 ^ab^	6.84 ± 0.1 ^ab^	6.84 ± 0.1 ^ab^	6.83 ± 0.1 ^ab^	6.80 ± 0.1 ^b^			
pCO_2_ (mmHg)	C	60.7 ± 10.5 ^b^	71.2 ± 11.9 ^ab^	76.2 ± 10.7 ^ab^	80.2 ± 13.6 ^a^	79.3 ± 11.3 ^a^	79.7 ± 16.0 ^a^	84.9 ± 8.5 ^a^	0.0435	0.0001	0.0001
	F	58.3 ± 10.1	64.0 ± 10.2	65.9 ± 11.8	68.9 ± 9.1	67.4 ± 8.0	67.1 ± 9.8	70.6 ± 7.6			
pO_2_ (mmHg)	C	50.5 ± 10.2 ^d^	72.3 ± 22 ^cd^	90.4 ± 16.4 ^bcd^	85.8 ± 27.5 ^bcd^	116.7 ± 42.8 ^abc^	147.8 ± 69.6 ^a^	135.8 ± 51.0 ^ab^	0.0001	0.0001	0.0001
	F	55.6 ± 7.2 ^d^	87.7 ± 19.6 ^d^	144.3 ± 37.8 ^c^	180.8 ± 35.5 ^bc^	183.4 ± 34.2 ^b^	241.6 ± 19.2 ^a^	225.9 ± 21.7 ^a^			
HCO_3_ (mmol/L)	C	13.8 ± 4.2 ^a^	13.2 ± 3.8 ^a^	12.9 ± 3.8 ^b^	12.6 ± 3.8 ^b^	12.0 ± 3.8 ^b^	11.7 ± 4.0 ^b^	11.4 ± 3.6 ^b^	0.9822	0.0001	0.7517
	F	13.4 ± 4.0 ^a^	13.2 ± 4.2 ^a^	12.7 ± 4.1 ^b^	12.5 ± 3.9 ^b^	12.2 ± 3.9 ^b^	11.8 ± 3.9 ^b^	11.6 ± 3.7 ^b^			
sO_2_ (mmHg)	C	59.2 ± 14.4 ^b^	71.4 ± 16.9 ^ab^	83.0 ± 8.2 ^a^	77.2 ± 11.6 ^ab^	83.3 ± 19.5 ^a^	86.2 ± 15.8 ^a^	86.5 ± 14.5 ^a^	0.0083	0.0001	0.2668
	F	67.1 ± 9.3 ^c^	84.2 ± 9.0 ^b^	94.4 ± 4.7 ^a^	97.5 ± 1.5 ^a^	97.2 ± 2.7 ^a^	98.9 ± 0.3 ^a^	98.7 ± 0.5 ^a^			

* *p* < 0.05 indicates overall difference between the tested blood bags. Different lowercase letters on the line indicate a difference between times through Tukey’s paired comparison test. pH: hydrogen potential; pO_2_: partial oxygen pressure; pCO_2_: partial carbon dioxide pressure; sO_2_: saturation of oxygen; HCO_3_: bicarbonate. D: day. Treat × Time: treatment and time interaction.

**Table 3:** Mean values and standard deviations of the biochemical variables of the cattle whole blood stored in CPD/SAG-M (C) and CPD/SAG-M with Filter (F) bags for 42 days.

Variables	Bags	Times							*p*		
		D0	D7	D14	D21	D28	D35	D42	Treatment *	Time	Treat × Time
Glucose (mg/dL)	C	600.6 ± 33.5 ^a^	600.5 ± 49.6 ^a^	564.0 ± 39.4 ^ab^	552.3 ± 30.6 ^ab^	544.4 ± 29.8 ^b^	534.5 ± 29.0 ^b^	545.9 ± 25.1 ^b^	0.6314	0.0001	0.0705
	F	597.0 ± 19.7 ^a^	589.9 ± 17.3 ^ab^	572.0 ± 30.9 ^abc^	565.8 ± 32.4 ^abc^	559.3 ± 23.5 ^bc^	552.2 ± 25.7 ^c^	546.8 ± 25.5 ^c^			
Cholesterol (mg/dL)	C	101.5 ± 16.1	107.8 ± 16.9	105.0 ± 16.4	104.3 ± 15.3	105.4 ± 15.9	107.3 ± 16.9	102.0 ± 15.2	0.7866	0.5481	0.0050
	F	103.8 ± 17.6	104.5 ± 16.0	103.2 ± 15.5	101.8 ± 15.2	104.3 ± 16.6	103.9 ± 16.2	100.1 ± 18.0			
Lactate (mg/dL)	C	80.4 ± 40.2 ^b^	99.4 ± 39.9 ^b^	104.1 ± 37.6 ^a^	111.7 ± 36.8 ^a^	107.6 ± 51.0 ^a^	127.2 ± 37.0 ^a^	126.2 ± 31.5 ^a^	0.7247	0.0001	0.1522
	F	81.0 ± 35.9 ^b^	90.7 ± 36.5 ^b^	104.7 ± 34.2 ^a^	98.3 ± 30.0 ^b^	106.8 ± 33.2 ^a^	107.6 ± 32.1 ^a^	113.1 ± 31.7 ^a^			
Potassium (mg/dL)	C	2.4 ± 0.2 ^e^	3.7 ± 0.3 ^d^	4.5 ± 0.4 ^c^	4.9 ± 0.4 ^bc^	5.2 ± 0.5 ^ab^	5.4 ± 0.5 ^ab^	5.7 ± 0.6 ^a^	0.1191	0.0001	0.0006
	F	2.4 ± 0.2 ^e^	3.4 ± 0.3 ^d^	4.1 ± 0.4 ^c^	4.6 ± 0.4 ^bc^	4.8 ± 0.5 ^ab^	5.0 ± 0.5 ^ab^	5.3 ± 0.6 ^a^			
Sodium (mg/dL)	C	143.7 ± 1.3 ^a^	141.4 ± 1.3 ^b^	141.2 ± 1.1 ^b^	140.6 ± 1.3 ^b^	140.9 ± 1.1 ^b^	140.5 ± 1.2 ^b^	140.4 ± 1.2 ^b^	0.6892	0.0001	0.0257
	F	143.8 ± 1.2 ^a^	142.1 ± 1.0 ^ab^	141.3 ± 1.5 ^b^	141.3 ± 1.6^b^	140.6 ± 1.3 ^b^	140.3 ± 1.2 ^b^	140.7 ± 1.2 ^b^			

* *p* < 0.05 indicates overall difference between the tested blood bags. Different lowercase letters on the line indicate a difference between times and through Tukey’s paired comparison test. D: day. Treat × Time: treatment and time interaction.
